# Molecular Bases and Phenotypic Determinants of Aromatase Excess Syndrome

**DOI:** 10.1155/2012/584807

**Published:** 2012-01-26

**Authors:** Maki Fukami, Makio Shozu, Tsutomu Ogata

**Affiliations:** ^1^Department of Molecular Endocrinology, National Research Institute for Child Health and Development, 2-10-1 Ohkura, Setagaya, Tokyo 157-8535, Japan; ^2^Department of Reproductive Medicine, Graduate School of Medicine, Chiba University, 1-8-1 Inohana, Chuo-ku, Chiba City 206-8670, Japan; ^3^Department of Pediatrics, Hamamatsu University School of Medicine, 1-20-1 Handayama, Higashi-ku, Shizuoka, Hamamatsu 431-3192, Japan

## Abstract

Aromatase excess syndrome (AEXS) is a rare autosomal dominant disorder characterized by gynecomastia. This condition is caused by overexpression of *CYP19A1* encoding aromatase, and three types of cryptic genomic rearrangement around *CYP19A1*, that is, duplications, deletions, and inversions, have been identified in AEXS. Duplications appear to have caused *CYP19A1* overexpression because of an increased number of physiological promoters, whereas deletions and inversions would have induced wide *CYP19A1* expression due to the formation of chimeric genes consisting of a noncoding exon(s) of a neighboring gene and *CYP19A1* coding exons. Genotype-phenotype analysis implies that phenotypic severity of AEXS is primarily determined by the expression pattern of *CYP19A1* and the chimeric genes and by the structural property of the fused exons with a promoter function (i.e., the presence or the absence of a natural translation start codon). These results provide novel information about molecular mechanisms of human genetic disorders and biological function of estrogens.

## 1. Introduction

Aromatase encoded by *CYP19A1* is a cytochrome P450 enzyme that plays a key role in estrogen biosynthesis [1]. It catalyzes the conversion of Δ^4^-androstendione into estrone (E_1_) and that of testosterone (T) into estradiol (E_2_) in the placenta and ovary as well as in other tissues such as the fat, skin, bone, and brain [1].

Overexpression of *CYP19A1* causes a rare autosomal dominant disorder referred to as aromatase excess syndrome (AEXS, OMIM no. 139300) [2–8]. AEXS is characterized by pre- or peripubertal onset gynecomastia, gonadal dysfunction, advanced bone age from childhood to pubertal period, and short adult height in affected males [2–8]. In particular, gynecomastia is a salient feature in AEXS, and, therefore, this condition is also known as hereditary gynecomastia or familial gynecomastia [5]. Affected females may also show several clinical features such as macromastia, precocious puberty, irregular menses, and short adult height [5, 6, 8].

Recently, three types of cryptic genomic rearrangements around *CYP19A1* have been identified in 23 male patients with AEXS [2–4]. The results provide useful implications not only for the clarification of underlying mechanisms but also for the identification of phenotypic determinants. Here, we review the current knowledge about AEXS. 

## 2. The Aromatase Gene (*CYP19A1*)

**Figure 1:**
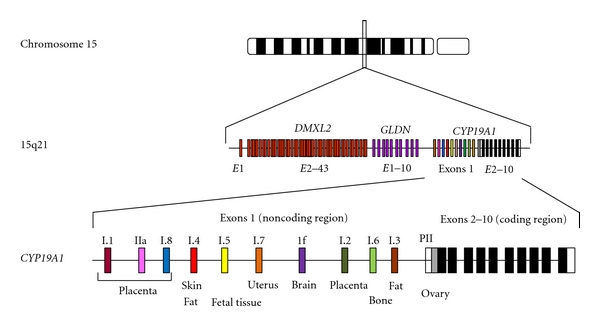
Simplified schematic representation indicating the genomic structure of *CYP19A1*. *CYP19A1 *is located on 15q21.2 adjacent to *DMXL2* and *GLDN* and consists of at least 11 noncoding exons 1 and nine coding exons 2–10 [9, 10]. Each exon 1 is accompanied by a tissue-specific promoter and is spliced alternatively onto a common splice acceptor site at exon 2 [9–13].

*CYP19A1* encoding aromatase is located on 15q21.2 adjacent to *DMXL2* and *GLDN* (Figure 1) [3, 9]. It spans ~123 kb and consists of at least 11 noncoding exons 1 and nine coding exons 2–10 [9–12]. Each exon 1 is accompanied by a tissue-specific promoter and is spliced alternatively onto a common splice acceptor site at exon 2, although some transcripts are known to contain two of the exons 1 probably due to a splice error [9–11]. Transcription of *CYP19A1* appears to be tightly regulated by alternative usage of the multiple promoters [9–13]. Actually, *CYP19A1* is strongly expressed in the placenta and moderately expressed in the ovary, whereas it is only weakly expressed in a rather limited number of tissues including skin, fat, and hypothalamus [4, 13]. Of the 11 noncoding exons 1, exon I.4 seems to play a critical role in the regulation of estrogen biosynthesis in males, because this exon contains the major promoter for extragonadal tissues [9, 10].

## 3. Molecular Bases of AEXS

A family with dominantly transmitted gynecomastia of pre-pubertal onset was first described in 1962 by Wallach and Garcia [14]. After this initial report, several cases have been described [5–8, 15]. Laboratory examinations of the affected males revealed markedly elevated serum estrogen values and estrogen/androgen ratios and significantly increased aromatase activity in fibroblasts and lymphocytes [5–8, 15]. Linkage analyses in two families indicated a close association between *CYP19A1*-flanking polymorphic markers and the disease phenotype [5, 6]. Thus, the condition was assumed to be caused by gain-of-function mutations of *CYP19A1, *and, therefore, the name of AEXS was coined for this condition [7, 8]. However, since direct sequencing and Southern blotting analysis failed to detect mutations or copy number abnormalities in the coding region of *CYP19A1 *[5, 6], the molecular basis of this entity remained elusive until recently.

In 2003, Shozu et al. reported a father-son pair and a sporadic case with AEXS in whom they identified heterozygous chromosomal inversions of the chromosome 15 [2]. Subsequently, Demura et al. performed detailed molecular studies for these cases and additional two cases and characterized four types of inversions affecting the 5′ region of *CYP19A1* [3]. Each inversion has resulted in the formation of a chimeric gene consisting of *CYP19A1* coding exons and exon 1 of the widely expressed neighboring genes, that is, *CGNL1*, *TMOD3*, *MAPK6,* and *TLN2*. These data imply that overexpression of *CYP19A1* in the inversion-positive cases are caused by cryptic usage of constitutively active promoters. Consistent with this, *in silico* analysis revealed the presence of promoter-compatible sequences around exon 1 of *CGN1*, *TMOD3*, and *MAPK6 *in multiple cell types, although such sequences remain to be identified for noncoding exons of *TLN2* [4].

**Figure 2:**
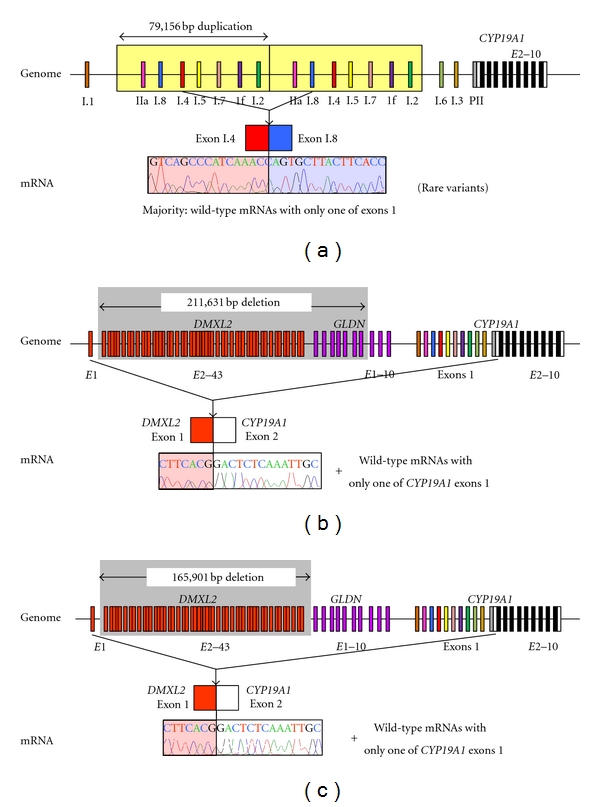
Schematic representation of duplications and deletions identified in patients with AEXS. (a) the tandem duplication of families A and B [4]. Genome: the duplication (yellow boxes) includes seven of the 11 noncoding exons 1 of *CYP19A1*. mRNA: the sequence of a rare transcript is shown. The 3′-end of exon I.4 is connected with the 5′-end of exon I.8. (b) The deletion of family C [4]. Genome: the deletion (a gray area) includes exons 2–43 of *DMXL2* and exons 5–10 of *GLDN*. mRNA: The sequence of a rare chimeric gene transcript is shown. *DMXL2* exon 1 consisting of a noncoding region and a coding region is spliced onto the common acceptor site of *CYP19A1* exon 2. (c) The deletion of families D–F [4]. Genome: the deletion (a gray area) includes exons 2–43 of *DMXL2*. mRNA: the sequence of a rare chimeric gene transcript is delineated. The mRNA structure is the same as that detected in family C.

We recently studied 18 males from six families with AEXS (families A–F) and identified three types of heterozygous cryptic genomic rearrangements in the upstream region of the *CYP19A1* coding exons (Figure 2) [4]. In families A and B, we identified the same 79,156 bp tandem duplication encompassing seven of the 11 noncoding exons 1 of *CYP19A1.* Notably, this duplication includes exon I.4 that functions as a major promoter for extragonadal tissues such as fat and skin; therefore, *CYP19A1* overexpression in these families would be explained by increasing the number of this promoter. Indeed, RT-PCR analysis detected a splice variant consisting of exon I.4 at the 5′ side and exon I.8 at the 3′ side in lymphoblastoid cell lines and skin fibroblasts of the patients, indicating that the duplicated exon I.4 at the distal nonphysiological position actually functions as transcription start sites. In family C, we identified a 211,631 bp deletion affecting exons 2–43 of *DMXL2* and exons 5–10 of *GLDN*. This deletion appears to have caused *CYP19A1* overexpression because of cryptic usage of *DMXL2* exon 1 as an extra transcription start site for *CYP19A1*. Indeed, RT-PCR revealed the presence of chimeric mRNA clones consisting of* DMXL2* exon 1 and *CYP19A1* exon 2, supporting the notion that aberrant splicing has occurred between these two exons. Such *DMXL2*/*CYP19A1* chimeric mRNA accounted for 2–5% of *CYP19A1*-containing transcripts from skin fibroblasts. In families D–F, we identified an identical 165,901 bp deletion including exons 2–43 of* DMXL2*. RT-PCR identified the same chimeric mRNA as that detected in family C. 

**Figure 3:**
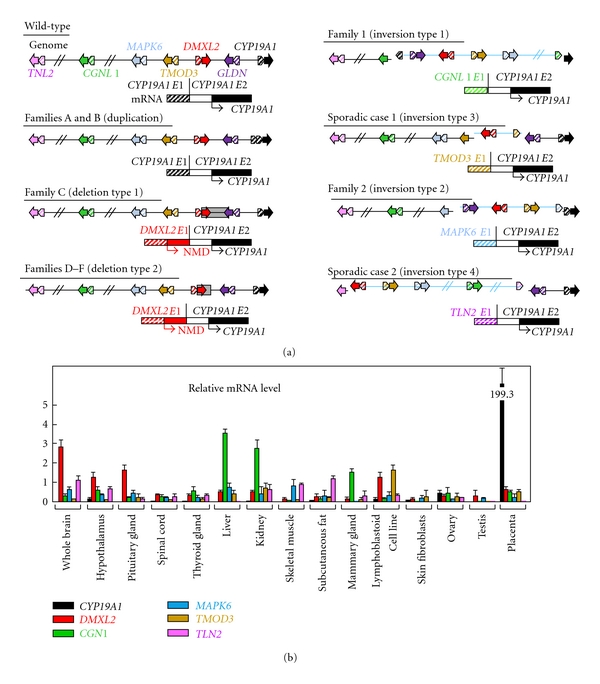
Structural and functional properties of the fused exons. (a) Schematic representation of the rearranged genome and mRNA structures. The white and the black boxes of *CYP19A1* exon 2 show untranslated region and coding region, respectively. For genome, the striped and the painted arrows indicate noncoding and coding exons, respectively (5′→3′). The inverted genomic regions are delineated in blue lines. For mRNA, colored striped boxes represent noncoding regions of each gene. The *DMXL2-CYP19A1* chimeric mRNA has two translation initiation codons and therefore is destined to produce not only *CYP19A1* protein but also a 47 amino acid protein which is predicted to undergo nonsense-mediated mRNA decay (NMD). The deletion and the inversion types are associated with heterozygous impairment of neighboring genes (deletion or disconnection between noncoding exon(s) and the following coding exons). The inversion subtype 1 is accompanied by inversion of eight of the 11 *CYP19A1* exons 1, and the inversion subtype 2 is associated with inversion of the placenta-specific *CYP19A1* exon I.1. (b) Expression patterns of *CYP19A1* and the five neighboring genes involved in the chimeric gene formation [4]. Relative mRNA levels against *TBP* in normal human tissues are shown.

Collectively, three types of genomic rearrangements on 15q21 have been identified in AEXS to date, namely, inversion type (four subtypes), duplication type, and deletion type (two subtypes) (Figure 3(a)) [2–4]. In this regard, sequence analyses for the breakpoints have indicated that (1) inversion types are formed by a repeat sequence-mediated nonallelic intrachromosomal or interchromosomal recombination or by a replication-based mechanism of fork stalling and template switching (FoSTeS) that occurs in the absence of repeat sequences and is often associated with microhomology [16], (2) duplication type is generated by FoSTeS, and (3) deletions are produced by nonhomologous end joining that takes place between nonhomologous sequences and is frequently accompanied by an insertion of a short segment at the fusion point or by a nonallelic recombination [16]. Thus, it appears that genomic sequence around *CYP19A1* harbors particular motifs that are vulnerable to replication- and recombination-mediated errors. The results provide novel mechanisms of gain-of-function mutations leading to human diseases. 

## 4. Clinical Features of AEXS

To date, a total of 23 male cases from 10 families have been reported to have molecularly confirmed AEXS (Table 1, Figure 3(a)) [2–4]. They exhibited pre- or peripubertal onset gynecomastia, small testes with fairly preserved masculinization, obvious or relative tall stature in childhood and grossly normal or apparent short stature in adulthood, and age-appropriate or variably advanced bone ages. Blood endocrine studies revealed markedly elevated E_1_ values and E_2_/T ratios in all cases examined and normal or variably elevated E_2_ values. In addition, Δ^4^-androstenedione, T, and dihydrotestosterone values were low or normal, and human chorionic gonadotropin (hCG) test indicated normal T responses. Notably, LH values were grossly normal at the baseline and variably responded to GnRH stimulation, whereas FSH values were low at the baseline and poorly responded to GnRH stimulation even after preceding GnRH priming, in all cases examined.

The severity of such clinical phenotypes is primarily dependent on the underlying mechanisms (Table 1). They are obviously mild in the duplication type, moderate in the deletion type, and severe in the inversion type, except for serum FSH values that remain suppressed irrespective of the underlying mechanisms. Likewise, gynecomastia has been reported to be ameliorated with 1 mg/day of aromatase inhibitor (anastrozole) in the duplication and the deletion types and with 2–4 mg/day of anastrozole in the inversion type [4].

## 5. Expression Pattern of *CYP19A1* and the Chimeric Genes as One Phenotypic Determinant

Phenotypic severity is much milder in the duplication type than in the deletion and the inversion types. This would be explained by the tissue expression pattern of *CYP19A1* and the chimeric genes. Indeed, RT-PCR analysis using normal human tissue samples revealed that *CYP19A1* is expressed only in a limited number of tissues such as placenta, ovary, skin, and fat, while the five genes involved in the formation of chimeric genes are widely expressed with some degree of variation (Figure 3(b)). Therefore, it is likely that the duplication types would simply increase *CYP19A1 *transcription in native *CYP19A1*-expressing tissues, whereas the deletion and the inversion types lead to *CYP19A1* overexpression in a range of tissues, because expression patterns of chimeric genes are predicted to follow those of the original genes. Furthermore, it is also likely that the native *CYP19A1* promoter is subject to negative feedback by elevated estrogens [17], whereas such negative feedback effect by estrogen is weak or even absent for the chimeric genes in the deletion and the inversion types.

## 6. Structural Property of the Fused Exons as Another Phenotypic Determinant

Phenotypic severity is also milder in the deletion type than in the inversion types, despite a similar wide expression pattern of genes involved in the chimeric gene formation (Table 1, Figure 3(b)). In this context, it is noteworthy that a translation start codon and a following coding region are present on exon 1 of *DMXL2* of the deletion type but not on exons 1 of the chimeric genes of the inversion types (Figure 3(a)). Thus, it is likely that *DMXL2*/*CYP19A1* chimeric mRNAs transcribed by the *DMXL2* promoter preferentially recognize the natural start codon on *DMXL2* exon 1 and undergo nonsense-mediated mRNA decay and that rather exceptional chimeric mRNAs, which recognize the start codon on* CYP19A1* exon 2, are transcribed into CYP19A1 protein. By contrast, such a phenomenon would not be postulated for the inversion-mediated chimeric mRNAs. Consistent with this, it has been shown that the *DMXL2*/*CYP19A1* chimeric mRNA is present only in 2–5% of *CYP19A1*-containing transcripts from skin fibroblasts, whereas the *CGNL1*/*CYP19A1* chimeric mRNA and the *TMOD3*/*CYP19A1* chimeric mRNA account for 89–100% and 80% of transcripts from skin fibroblasts, respectively [2, 4].

In addition, the genomic structure caused by the rearrangements would affect efficiency of splicing between noncoding exon(s) of neighboring genes and *CYP19A1* exon 2. For example, in the inversion subtype 1, the physical distance between *CGNL1 *exon 1 and *CYP19A1* exon 2 is short, and, while a splice competition may be possible between exon 1 of neighboring genes and original *CYP19A1 *exons 1, eight of 11 *CYP19A1* exons 1 including exon I.4 have been disconnected from *CYP19A1* coding exons by inversion (Figure 3(a)). This may also enhance the splicing efficiency between *CGNL1* exon 1 and *CYP19A1* exon 2 and thereby lead to relatively severe overexpression of the *CGNL1-CYP19A1* chimeric gene, although this hypothesis would not be applicable for other chimeric genes.

## 7. Implication for the Hypothalamus-Pituitary-Gonadal Axis Function

It is notable that a similar degree of FSH-dominant hypogonadotropic hypogonadism is observed in the three types, although E_1_ and E_2_ values and E_2_/T ratios are much higher in the inversion type than in the duplication and deletion types (Table 1). In particular, FSH was severely suppressed even after GnRH priming in the duplication type [4]. This implies that a relatively mild excess of circulatory estrogens can exert a strong negative feedback effect on FSH secretion primarily at the pituitary. This would be consistent with the results of animal studies that show strong inhibitory effect of E_2_ on transcription of FSH beta-subunit gene in the pituitary cells and almost negligible effect on synthesis of LH beta-subunit and secretion of LH [18, 19]. In this regard, while T responses to hCG stimulation are normal in the duplication and the deletion types and somewhat low in the inversion type, this would be consistent with fairly preserved LH secretion in the three types and markedly increased estrogen values in the inversion type. In addition, whereas fertility and spermatogenesis are normally preserved in the three types, this would be explained by the FSH-dominant hypogonadotropic hypogonadism, because FSH plays only a minor role in male fertility (spermatogenesis) [20].

## 8. Conclusions

Current studies argue that AEXS is caused by overexpression of *CYP19A1* due to three different types of cryptic genomic rearrangements including duplications, deletions, and inversions. It seems that transcriptional activity and structural property of the fused promoter constitutes the underlying factor for the clinical variability in most features of AEXS except for FSH-dominant hypogonadotropic hypogonadism. Thus, AEXS represents a novel model for gain-of-function mutation leading to human genetic disorders.

## Figures and Tables

**Table 1a:** (a) (a)

Family		Family A					Family B		Family C				Family D					Family E	
Mutation types		Duplication					Duplication		Deletion				Deletion					Deletion	
The promoter involved in *CYP19A1 *overexpression		*CYP19A1*					*CYP19A1*		*CYP19A1*				*DMXL2*					*DMXL2*	
Case		Case 1	Case 2		Case 3		Case 4		Case 5		Case 6		Case 7	Case 8		Case 9		Case 10	
Age at examination (year)		66	15		20		15		15		13		42	9		12		13	

<Phenotypic findings>																			
Gynecomastia (tanner breast stage)		**2**	**2**		**2**		**3**		**4**		**4**		**4**	**3**		**4**		**4**	
Onset of gynecomastia (year)		**13**	**13**		**10**		**11**		**12**		**11**		**11**	**7**		**9**		**10**	
Mastectomy (year)		No	**Yes (15)**		No		**Yes (15)**		**Yes (15)**		**Yes (13)**		No	No		**Yes (12)**		**Yes (13)**	
Testis (ml)		N.E.	**12**		**12**		**12**		**12**		12		N.E.	3		12		20	
Pubic hair (tanner stage)		N.E.	2-3		4		5		4		3		N.E.	1		3		4	
Facial hair		Normal	Scarce		**Scarce**		Normal		Absent		Absent		N.E.	Absent		Absent		Absent	
Height (SDS)^a^		−1.2	−0.3		+0.4		+0.8		−2.0		−1.0		−1.6	**+2.7**		±0		+1.8	
Bone age (year)^b^		N.E.	N.E.		N.E.		16.0		16.0		13.5		N.E.	**13.0**		**15.0**		**17.0**	
Fertility (spermatogenesis)		Yes	?		(Yes)^h^		?		?		?		Yes	?		?		?	

<Endocrine findings>^c^		B	B	S	B	S	B	S	B	S	B	S	B	B	S	B	S	B	S
<At Dx>	Stimulus																		
LH (mIU/mL)	GnRH^e^	3.8	2.3	**14.3**	2.1	**17.0**	2.4	29.4	1.9	40.6	1.8	69.2		1.1	11.5	**0.6**	39.5	6.7	14.8
LH (mIU/mL)	GnRH (after priming)^f^		1.8	**9.5**	**1.3**	**10.7**													
FSH (mIU/mL)	GnRH^e^	**1.7**	**3.1**	**5.3**	**<0.5**	**1.2**	**0.9**	**2.4**	**1.4**	**4.2**	**2.0**	**7.8**		**3.2**	**6.6**	**0.6**	**2.9**	**0.7**	**1.0**
FSH (mIU/mL)	GnRH (after priming)^f^		**2.6**	**3.2**	**<0.5**	**0.9**													
Prolactin (ng/ml)			4.3		5.3				8.2		9.1			11.3		18.8			
Δ^4^A (ng/mL)		**0.5**			**1.1**		**1.2**						**0.6**			**0.7**		**2.4**	2.9
T (ng/mL)	hCG^g^	2.9	**1.6**		**2.2**		4.0		**2.6**	7.2	**1.4**	7.9		**0.6**	3.6	**2.4**		3.2	9.7
DHT (ng/mL)		0.4			0.2													0.4	1.2
Inhibin B (pg/mL)		**61.6**			**74.6**		**83.5**		**75.2**										
E_1_ (pg/mL)		157			120		124						57			63		53	
E_2_ (pg/mL)		29	15		22		59		56		38		24	19		25		58	
E_2_/T ratio (×10^3^)		10.0	9.4		10.0		14.8		21.5		27.1			31.7		10.4		18.1	

**Table 1b:** (b) (b)

Family		Family F									Family G		Family H		Sporadic	
Mutation types		Deletion									Inversion		Inversion		Inversion	
The promoter involved in *CYP19A1 *overexpression		*DMXL2*									*CGNL1*		*MAPK6*		*TMOD3*	*TLN2*
Case		Case 11	Case 12	Case 13	Case 14	Case 15	Case 16	Case 17		Case 18	Case 19	Case 20	Case 21^j^		Case 22	Case 23
Age at examination (year)		69	35	44	45	9	8	13		10	35	7	13		17	36

<Phenotypic findings>																
Gynecomastia (tanner breast stage)		**Yes** ^ i^	**Yes** ^ i^	**Yes** ^ i^	**Yes** ^ i^	**2**	**3**	**3**		**3**	**Yes**	**3**	**5**		N.E.	**Yes**
Onset of gynecomastia (year)		?	?	?	?	**8**	**8**	**11**		**10**	**5**	**5**	**8**		**7**	?
Mastectomy (year)		**Yes** ^ i^	**Yes** ^ i^	**Yes** ^ i^	**Yes** ^ i^	No	No	**Yes (?)**		**Yes (?)**	**Yes (16)**	No	**Yes (?)**		**Yes (?)**	**Yes (19)**
Testis (ml)		N.E.	N.E.	N.E.	N.E.	2	1.5	**2**		2	N.E.	N.E.	N.E.		Normal	N.E.
Pubic hair (tanner stage)		N.E.	N.E.	N.E.	N.E.	1	1	2		1	Normal	1	**2-3 **(at 21.0)		N.E.	N.E.
Facial hair		N.E.	N.E.	N.E.	N.E.	Absent	Absent	Absent		Absent	**Absent**	Absent	N.E.		**Scarce**	N.E.
Height (SDS)^a^		N.E.	~−1.5	~−1.5	~−1.5	+1.4	N.E.	**+2.0**		**+2.4**	**Short**	**>+2.5**	−1.6 (at 21.0)		**Short**	N.E.
Bone age (year)^b^		N.E.	N.E.	N.E.	N.E.	**12.5**	**13.0**	**15.0**		**14.5 ** **(at 12.5)**	N.E.	**13.0 ** **(at 5.5)**	**17.0**		N.E.	N.E.
Fertility (spermatogenesis)		Yes	Yes	Yes	Yes	?	?	?		?	Yes	?	?		?	?

<Endocrine findings>^c^		B	B	B	B	B	B	B	S	B	B	B	B	S	B	
<At Dx>	Stimulus															
LH (mIU/mL)	GnRH^e^	**0.2**	3.5	1.7	3.0	0.2	<0.1	2.6	6.3	1.5	1.7	0.1	2.6	**10.0**	4.3	
LH (mIU/mL)	GnRH (after priming)^f^															
FSH (mIU/mL)	GnRH^e^	**1.4**	**2.3**	**0.8**	**0.8**	1.4	0.5	**0.8**	**1.2**	**1.2**	**1.5**	0.3	**<0.1**	**<0.1**	**2.7**	
FSH (mIU/mL)	GnRH (after priming)^f^															
Prolactin (ng/ml)																
Δ^4^A (ng/mL)		1.4	**0.4**	1.7	**0.5**	0.3	<0.3	**0.9**	1.5	1.3	**0.8**	0.3	2.4	**0.9**		
T (ng/mL)	hCG^g^	**2.6**	**2.5**	**2.1**	**2.5**	<0.1	<0.1	**2.7**	9.2	**2.7**	3.2	<0.1	**1.2**	3.8	**2.3**	
DHT (ng/mL)													0.2	0.5		
Inhibin B (pg/mL)																
E_1_ (pg/mL)		32	34	59	34	26	41	77		86	903	119	544		556	
E_2_ (pg/mL)		10	19	24	31	11	7	25		40	223	15	178		392	
E_2_/T ratio (×10^3^)		3.8	7.6	11.4	12.4			9.3		14.8	69.6		148.3		170.4	

SDS: standard deviation score; Dx: diagnosis; Tx: therapy; LH: luteinizing hormone; FSH: follicle stimulating hormone; Δ^4^A: androstenedione; T: testosterone; DHT: dihydrotestosterone;

E_1_: estrone; E_2_: estradiol; GnRH: gonadotropin-releasing hormone; hCG: human chorionic gonadotropin; N.E.: not examined; B: basal; and S: stimulated.

Abnormal clinical findings are boldfaced.

Abnormally low hormone values are boldfaced, and abnormally high hormone values are underlined.

^a^Evaluated by age- and ethnicity-matched growth references; heights ≥+2.0 SD or below ≤−2.0 SD were regarded as abnormal.

^b^Assessed by the Tanner-Whitehouse 2 method standardized for Japanese or by the Greulich-Pyle method for Caucasians; bone age was assessed as advanced when it was accelerated a year or more.

^c^Evaluated by age-matched male reference data, except for inhibin B and E_1_ that have been compared with data from 19 adult males.

^d^Treated with aromatase inhibitors (anastrozole).

^e^GnRH 100 *μ*g/m^2^ (max. 100 *μ*g) bolus i.v.; blood sampling at 0, 30, 60, 90, and 120 minutes.

^f^GnRH test after priming with GnRH 100 *μ*g i.m. for 5 consecutive days.

^g^hCG 3000 IU/m^2^ (max 5000 IU) i.m. for 3 consecutive days; blood sampling on days 1 and 4.

^h^Although Case 3 has not yet fathered a child, he has normal spermatogenesis with semen volume of 2.5 ml (reference value: >2 ml), sperm count of 105 × 10^6^/ml (>20 × 10^6^/ml), total sperm count of 262.5 × 10^6^ (>40 × 10^6^), motile cells of 70% (>50%), and normal morphological sperms 77% (>30%).

^i^These four patients allegedly had gynecomastia that required mastectomy (age unknown).

^j^The sister has macromastia, large uterus, and irregular menses; the parental phenotype has not been described.

The conversion factor to the SI unit: LH 1.0 (IU/L), FSH 1.0 (IU/L), E_1_ 3.699 (pmol/L), E_2_ 3.671 (pmol/L), Δ^4^A 3.492 (nmol/L), and T 3.467 (nmol/L).
